# BjPro-7a, A Proline-Rich Peptide from *Bothrops jararaca* Venom, Reverses MPP⁺-Induced Locomotor Deficits and Rescues Mitochondrial, Redox, and Synaptic Proteomic Pathways in a Zebrafish Model of Parkinsonism

**DOI:** 10.1007/s11064-026-04835-2

**Published:** 2026-07-13

**Authors:** Adolfo Luis Almeida Maleski, Felipe Assumpção da Cunha e Silva, Bruno Fiorelini Pereira, Carlos Alberto-Silva

**Affiliations:** 1 Experimental Morphophysiology Laboratory, Natural and Humanities Sciences Center (CCNH), Universidade Federal do ABC (UFABC), São Bernardo do Campo, 09606-070 SP Brazil; 2 Experimental Danio rerio Facility (BEDAR), Universidade Federal de São Paulo – campus Diadema, Diadema, São Paulo, Brazil; 3 Department of Pharmaceutical Sciences, Universidade Federal de São Paulo – campus Diadema, Diadema, São Paulo, Brazil

**Keywords:** Behavioral phenotyping, Label-free proteomics, Mitochondrial homeostasis, Parkinsonism, Venom-derived peptide, Zebrafish model

## Abstract

Parkinson’s disease (PD) and related Parkinsonian conditions involve progressive disruption of mitochondrial homeostasis, redox balance, synaptic function, and proteostasis. Available therapies remain predominantly symptomatic, motivating the search for multitarget compounds acting across stress-sensitive pathways. BjPro-7a (pEDGPIPP), a proline-rich oligopeptide from *Bothrops jararaca* venom, exerts cytoprotective effects under oxidative stress, but its activity in an in vivo Parkinsonian-like model is unknown. Here we investigated whether BjPro-7a attenuates the behavioral and proteomic disturbances induced by 1-methyl-4-phenylpyridinium (MPP^+^) in zebrafish larvae. Larvae were exposed to 500 µM MPP^+^ and post-treated with 10 µM BjPro-7a, followed by assessment of basal locomotion, light/dark-evoked behavior, and label-free proteomic profiling. BjPro-7a markedly reversed the MPP^+^-induced hypolocomotor phenotype by increasing the total distance traveled, improving bout-related parameters, reducing inter-episode intervals, and restoring visually evoked responsiveness. Proteomic analysis revealed broad rescue-associated remodeling, more pronounced among proteins suppressed by MPP^+^ than among those elevated by intoxication. Functional enrichment and protein–protein interaction analyses indicated coordinated modulation of vesicle trafficking and synaptic organization, mitochondrial and bioenergetic function, redox homeostasis, and protein quality control. Representative rescue-associated proteins included VAMP2 and SNAP25A (synaptic), SDHA and UQCRFS1 (mitochondrial), G6PD and PRDX3 (redox), and PSMC5 and STIP1 (proteostasis). Together, these findings show that BjPro-7a attenuates MPP^+^-induced dysfunction and promotes systems-level proteomic remodeling consistent with rescue in zebrafish larvae. These data support BjPro-7a as a promising candidate for future therapeutic studies in Parkinsonian-like neurotoxicity.

**Graphical Abstract:**

**Figure:**
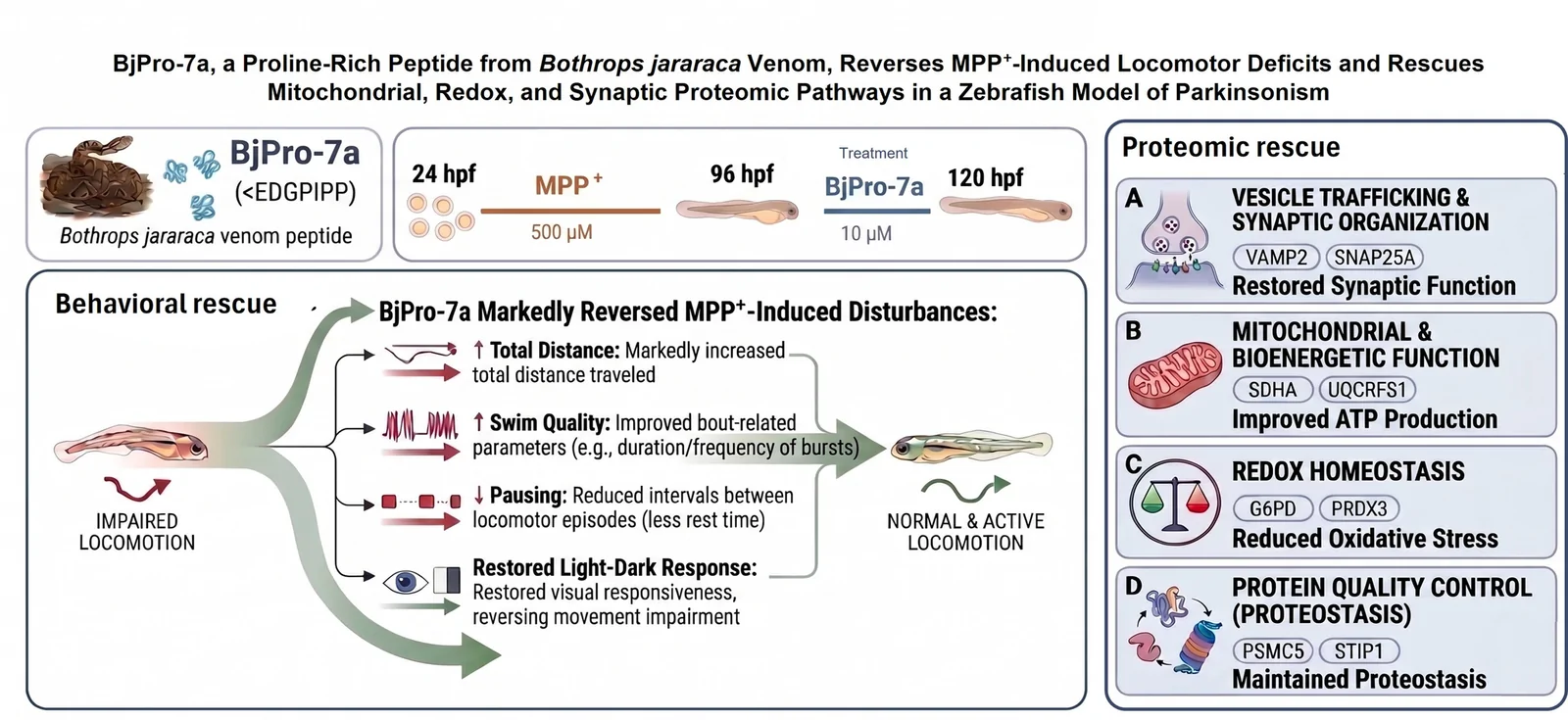

## Introduction

Neurodegenerative diseases comprise a heterogeneous group of disorders that differ in their predominant clinical phenotypes but frequently converge on a limited set of early pathogenic processes. Long before full phenotypic divergence becomes evident, disorders such as Parkinson’s disease (PD), Alzheimer’s disease, Huntington’s disease, and amyotrophic lateral sclerosis often share molecular alterations involving mitochondrial dysfunction, oxidative stress, impaired proteostasis, and synaptic failure [1–3]. These disturbances are not simply downstream consequences of neuronal loss. Rather, they are increasingly recognized as central drivers of neuronal vulnerability and progressive functional decline [1, 4, 5]. This convergence is particularly important because it suggests that effective interventions may need to stabilize several stress-sensitive systems simultaneously rather than target a single isolated lesion.

Within this broader context, PD remains one of the most clinically relevant and therapeutically challenging neurodegenerative disorders. Although classically defined by motor manifestations such as bradykinesia, rigidity, resting tremor, and postural instability, PD is now understood as a systems-level disorder involving mitochondrial bioenergetic failure, oxidative imbalance, altered vesicle trafficking, defective proteostasis, and progressive synaptic dysfunction [3–8]. Importantly, current treatments remain largely symptomatic. Dopaminergic replacement strategies can improve motor performance, but no available therapy has yet been conclusively shown to halt disease progression or reverse the underlying pathogenic state [7]. This gap between symptomatic benefit and true disease modification has reinforced interest in compounds capable of acting across multiple biological axes relevant to neuronal dysfunction.

In this scenario, animal venoms have emerged as particularly attractive sources of bioactive molecules with unusual pharmacological properties and high target selectivity. Venom-derived compounds have already inspired successful therapeutics, and recent work increasingly highlights venom peptides as promising scaffolds for disorders in which conventional small molecules remain insufficient [9–11]. Their appeal lies not only in their chemical diversity, but also in their capacity to interact with receptors, ion channels, enzymes, and signaling pathways directly linked to excitability, survival, and adaptive stress responses [12, 13]. This is especially relevant in neurodegenerative conditions, in which a multitarget profile may be more advantageous than narrowly restricted pharmacology.

Among snake venoms, *Bothrops jararaca* has yielded a particularly interesting class of proline-rich oligopeptides with biological actions extending beyond their classical cardiovascular relevance. Work from our group has progressively shown that these peptides can exert protective effects in oxidative stress-related models. In SH-SY5Y cells, BjPro-7a and related BjPro peptides increased cell viability and reduced oxidative stress markers under H_2_O_2_ challenge [14]. In PC12 cells, the peptide fraction from *B. jararaca* venom protected against oxidative-stress-induced loss of cell integrity and metabolic activity, whereas the same fraction failed to protect astrocyte-like C6 cells under equivalent conditions, suggesting that the response may be cell-type dependent rather than nonspecific [15]. More recently, structurally related proline-rich decapeptides were shown to differ substantially in both neuroprotective profile and effects on argininosuccinate synthase/L-arginine-related pathways, reinforcing the view that these peptides should not be treated as a generic class, but rather as molecules with distinct mechanistic signatures [16]. In parallel, our group demonstrated that the peptide fraction from *B. jararaca* also exerts in vivo protective effects in zebrafish subjected to oxidative stress, whereas a related fraction from *Daboia siamensis* did not reproduce this response under the same treatment conditions [17]. Together, these observations provide a strong rationale for investigating BjPro-7a in a Parkinsonian-like setting.

BjPro-7a itself is especially attractive because its biological profile suggests that it may influence neuronal function through more than one route. BjPro-7a acts as an agonist of the M1 muscarinic acetylcholine receptor (M1 mAChR) [18], and previous studies have shown that it rescues oxidative-stress-induced neurotoxicity in PC12 cells through a mechanism consistent with M1 receptor activation, restoring cell integrity, mitochondrial metabolism, and ROS-related alterations under H_2_O_2_ challenge [19]. In addition, behavioral studies in rodents indicated that BjPro-7a produces anxiolytic- and antidepressant-like effects while increasing locomotion and exploration, with evidence for the involvement of M1 receptor activation and catecholaminergic pathways [20]. Taken together, these findings suggest that BjPro-7a is not merely a cytoprotective molecule in simplified cell systems, but a peptide capable of engaging neural signaling pathways and influencing integrated functional outputs in vivo. More broadly, the therapeutic relevance of M1-directed signaling in neurodegenerative disorders has gained increasing support, particularly in the context of synaptic plasticity, neuronal excitability, and disease-modifying cholinergic strategies [21, 22].

Among currently available in vivo platforms, zebrafish have become particularly useful for studying candidate compounds in early neurodegenerative-like conditions. Their rapid development, optical accessibility, high-throughput compatibility, and quantifiable locomotor behavior make them especially suitable for evaluating toxicant-induced dysfunction and treatment responses [23–26]. In PD-oriented research, zebrafish also offer the advantage of allowing organism-level behavioral readouts to be directly linked with proteomic and systems-level molecular analyses [23–26]. This is particularly valuable when the goal is not only to detect neurotoxicity, but also to determine whether a candidate compound can reverse an already established dysfunctional state.

Within zebrafish-based PD modeling, the MPTP/MPP^+^ axis remains one of the best characterized toxin paradigms. MPP^+^ is widely used because of its close association with mitochondrial dysfunction, oxidative imbalance, and locomotor impairment [4, 6, 23–29]. In our previous study, chronic larval exposure to MPP^+^ generated a robust hypolocomotor phenotype together with proteomic remodeling enriched in mitochondrial, redox, proteostatic, and synaptic processes, thereby establishing a defined intoxication signature in zebrafish larvae [29]. This previous characterization is important for the present study because it allows the current question to be formulated more precisely: rather than asking whether MPP^+^ produces a relevant phenotype, the key issue becomes whether BjPro-7a can attenuate or reverse the functional and molecular disturbances already mapped in this model.

Based on this rationale, the aim of the present study was to investigate whether BjPro-7a can reverse the behavioral and proteomic alterations induced by MPP^+^ in zebrafish larvae. By combining locomotor phenotyping with label-free proteomic profiling and integrative systems-level analysis, we sought to determine whether BjPro-7a merely changes isolated readouts or instead promotes coordinated rescue of the core cellular systems disrupted by intoxication. In doing so, this study positions BjPro-7a within a broader effort to identify venom-derived peptides with genuine therapeutic potential in early Parkinsonian-like neurotoxicity.

## Materials and Methods

### Peptide and Reagents

BjPro-7a is a synthetic heptapeptide derived from *Bothrops jararaca* venom, corresponding to the sequence pGlu-Asp-Gly-Pro-Ile-Pro-Pro (pEDGPIPP), and was custom synthesized by GenOne Biotecnologia (Rio de Janeiro, Brazil). 1-Methyl-4-phenylpyridinium iodide (MPP^+^) was obtained from Sigma-Aldrich (St. Louis, MO, USA). E3 embryo medium was prepared using NaCl, KCl, CaCl_2_, and MgSO_4_. For protein extraction and quantification, urea, 1-S-octyl-beta-D-thioglucopyranoside (OG), Bradford reagent, and bovine serum albumin (BSA) were used, as in our previously standardized workflow [29].

### Zebrafish Maintenance, Breeding, and Ethics

Adult zebrafish of the Tübingen (TU) strain were housed and bred at the animal facility of the Federal University of São Paulo (Unifesp), São Paulo campus. All procedures involving animal care and experimental use were performed in accordance with the Brazilian National Council for the Control of Animal Experimentation guidelines (CONCEA, Normative Resolution No. 61/2023). The study protocols were reviewed and approved by the Institutional Ethics Committee for Animal Use at Unifesp (CEUA No. 5220291122). Animal welfare was assessed on a daily basis. Adult fish were maintained in 3 L polycarbonate tanks (Alesco^®^, São Paulo, Brazil) connected to a recirculating aquaculture system (Alesco^®^, São Paulo, Brazil), under standardized environmental conditions: pH 7.0 ± 0.5, conductivity of 6 µOsm, ammonia (0 ppm), nitrite (0 ppm), temperature of 28 ± 1 °C, and a 14:10 h light/dark cycle (OECD, 1992). Feeding was performed three times per day using commercial flake food (Alcon^®^ Colours; 42% protein), supplemented twice daily with *Artemia salina* nauplii provided *ad libitum* (Artemia salina do RN^®^).

### Experimental Design and MPP⁺/BjPro-7a Exposure Protocol

The experimental design comprised four groups: Control, MPP^+^, BjPro-7a, and MPP^+^/BjPro-7a. Only morphologically normal embryos and larvae, with no visible developmental abnormalities, were included in the experiments. At 24 h post-fertilization (hpf), embryos were randomly assigned to the four groups and maintained in E3 embryo medium at 28 °C under a 14:10 light: dark photoperiod. MPP^+^ was diluted in E3 embryo medium to a final concentration of 500 µM and administered from 1 to 5 days post-fertilization (dpf), following the intoxication protocol previously standardized by our group [24, 29].

BjPro-7a was used at a final concentration of 10 µM, selected based on previous in vitro studies from our group demonstrating effective protective activity of BjPro-7a at this concentration [14, 19]. In the MPP^+^/BjPro-7a group, larvae were initially exposed to MPP^+^ during the established intoxication period. After this exposure, the MPP^+^-containing medium was removed and replaced with fresh E3 medium containing BjPro-7a, and larvae remained under peptide treatment for an additional 24 h. In the BjPro-7a group, the same experimental timeline was followed, except that larvae were maintained in E3 medium without MPP^+^ during the initial period and were subsequently exposed to BjPro-7a alone for 24 h. Treatment solutions were replaced daily and contained the original concentration of the test compounds. Behavioral assays were performed with 12 larvae per group. For proteomic analysis, two biological replicates per group were prepared, each consisting of a pool of 30 larvae collected at 5 dpf (equivalent to 120 hpf, the same developmental time point used for behavioral assessment), following the same workflow adopted in our previously standardized study [29].

### Metabolic Activity

At the end of the treatment period (5 dpf), the larval medium from each experimental condition was replaced with E3 medium containing resazurin at a final concentration of 80 μmol·L⁻¹ [29]. After a 2-hour incubation period, the reduction of resazurin to resorufin was assessed by fluorescence measurement using a microplate reader (BioTek, Winooski, VT, USA), with excitation and emission wavelengths set at 530 and 590 nm, respectively.

### Behavioral Assessment

Behavioral analysis was performed at 120 hpf as a functional readout of MPP^+^-induced locomotor dysfunction and peptide-associated rescue. Before recording, larvae were gently transferred to fresh E3 medium to avoid carryover of treatment solutions. Larvae were individually placed in 24-well plates, one larva per well, containing 2 mL of fresh E3 embryo medium. Plates were positioned in a custom-designed behavioral tracking chamber under controlled environmental conditions. After a 15 min acclimation period in darkness, behavior was recorded for 180 s under alternating light/dark cycles consisting of 30 s light and 30 s dark, repeated three times [24, 29].

The following endpoints were analyzed: total distance traveled, mean velocity, distance per bout, bout interval, proportion of active/inactive time, number of bouts, bout duration, and locomotor profile over time. In the light/dark assay, shaded regions were used to indicate illuminated phases and facilitate interpretation of visually evoked responsiveness. Representative swimming trajectories and temporal locomotor traces were generated from the tracking dataset for figure presentation. Videos were analyzed in Fiji/ImageJ 2 (NIH, Bethesda, MD, USA), and extracted data were organized and plotted in GraphPad Prism 8 (GraphPad Software, San Diego, CA, USA) [30].

### Proteomic Sample Preparation

For label-free proteomic analysis, larval samples were collected at the experimental endpoint (5 dpf). Samples were processed according to the workflow previously standardized in our zebrafish MPP^+^ proteomic study [29]. Briefly, pooled larvae were dried in a SpeedVac concentrator (Savant SPD120, Thermo Fisher Scientific, Waltham, MA, USA) and resuspended in 100 µL of extraction buffer containing 150 mM Tris-HCl (pH 8.8), 8 M urea, and 0.5% OG. Samples were subjected to three ultrasonication cycles (1 min each at 30% amplitude, with 2 s intervals) on ice and then centrifuged at 10,000 × *g* for 10 min at 4 °C. Supernatants were collected, and proteins were precipitated with acetone: ethanol: formic acid (50:49.5:0.5, v/v/v), incubated at –20 °C for 3 h, and pelleted by centrifugation at 12,000 × *g* for 10 min at 4 °C. Pellets were resuspended in 50 µL of 150 mM Tris-HCl (pH 8.8).

Protein concentration was determined by the Bradford method using bovine serum albumin as standard. Aliquots were diluted 1:20 in deionized water, and 20 µL of each sample were loaded in duplicate into a 96-well microplate. Absorbance was measured at 595 nm in a SpectraMax Plus 384 microplate reader (Molecular Devices, San Jose, CA, USA) [29].

### LC–MS/MS Analysis

Proteomic analysis was performed on an Orbitrap Eclipse Tribrid mass spectrometer (Thermo Fisher Scientific, Bremen, Germany) coupled to a Dionex Ultimate 3000 RSLCnano system (Thermo Fisher Scientific, Germering, Germany). Approximately 1 µg of each digested peptide sample was injected onto a NanoEase M/Z Peptide BEH C18 analytical column (Waters Corporation, Milford, MA, USA; 130 Å, 1.7 μm, 75 μm × 250 mm) at a flow rate of 300 nL/min. Peptides were separated using a 90 min linear gradient from 4% to 50% acetonitrile in 0.1% formic acid. Full MS scans were acquired over *m/z* 375–1500 at a resolution of 120,000, with an automatic gain control target of 1 × 10^6^ and a maximum injection time of 100 ms. The most intense precursor ions were fragmented by higher-energy collisional dissociation using a normalized collision energy of 30%, an isolation window of 1.2 *m/z*, an automatic gain control target of 1 × 10^5^, and an MS/MS resolution of 15,000. LC–MS/MS acquisition followed the same analytical platform and settings adopted in our previous study [29].

### Proteomic Data Processing and Bioinformatics

Raw MS files were converted to mzXML format and analyzed using the Comet search engine against the *Danio rerio* UniProt reference proteome. Peptide-spectrum matches were validated with PeptideProphet using a false discovery rate threshold of ≤3%. Protein quantification was performed with the Xpress algorithm, and peptide intensities were aggregated at the protein level using a custom R script, following the workflow previously applied to our zebrafish MPP^+^ dataset [29]. Protein abundance matrices were then organized across the four experimental groups (Control, BjPro-7a, MPP^+^, and MPP^+^/BjPro-7a) for integrated comparison.

Three main comparisons were used for interpretation: MPP^+^ vs. Control, MPP^+^ vs. MPP^+^/BjPro-7a, and MPP^+^/BjPro-7a vs. Control. Rescue-associated proteins were defined as those altered in MPP^+^ vs. Control that shifted in the opposite direction in MPP^+^ vs. MPP^+^/BjPro-7a, with the peptide-treated group moving toward the control profile. Based on this logic, representative proteins were categorized as restored, normalized, or partially restored/attenuated, according to the directional framework summarized in Table 1. Within this framework, ‘restored’ refers to proteins decreased in MPP+ vs. Control that returned to levels approaching the control profile in MPP+/BjPro-7a; ‘normalized’ refers to proteins increased in MPP+ vs. Control that were reduced toward control levels in MPP+/BjPro-7a; and ‘partially restored/attenuated’ refers to proteins that showed a directional correction but did not fully return to the control-range. The BjPro-7a vs. Control comparison was also included to assess whether peptide treatment alone produced any detectable alteration in the proteomic profile relative to untreated larvae.

**Table 1:** Representative rescue-associated proteins modulated by BjPro-7a in MPP^+^-challenged zebrafish larvae

Gene symbol	Protein name	Alteration in MPP^+^ larvae	Effect of BjPro-7a in intoxicated larvae	Biological significance
VAMP2	Vesicle-associated membrane protein 2	Decreased	Restored	Synaptic vesicle trafficking and neurotransmitter release
SNAP25A	Synaptosome-associated protein 25a	Decreased	Restored	SNARE complex assembly and vesicle fusion
SYT5B	Synaptotagmin 5b	Increased	Normalized	Calcium-dependent vesicle dynamics
SLC25A11	Solute carrier family 25 member 11	Decreased	Restored	Mitochondrial metabolite transport
SDHA	Succinate dehydrogenase subunit A	Decreased	Restored	Complex II activity and oxidative metabolism
IDH2	Isocitrate dehydrogenase 2	Decreased	Restored	Tricarboxylic acid cycle and mitochondrial redox balance
UQCRFS1	Ubiquinol-cytochrome c reductase Rieske iron-sulfur subunit 1	Decreased	Restored	Complex III function and electron transport
COX6A1	Cytochrome c oxidase subunit 6A1	Decreased	Restored	Complex IV function and mitochondrial respiration
COX6C	Cytochrome c oxidase subunit 6 C	Increased	Normalized	Respiratory chain remodeling
UQCRQ	Ubiquinol-cytochrome c reductase subunit 8	Increased	Normalized	Complex III-associated mitochondrial adaptation
CS	Citrate synthase	Decreased	Partially restored/attenuated	Tricarboxylic acid cycle and mitochondrial metabolic competence
DLST	Dihydrolipoamide S-succinyltransferase	Decreased	Restored	2-oxoglutarate dehydrogenase complex and mitochondrial energy metabolism
TOMM7	Translocase of outer mitochondrial membrane 7	Altered	Partially restored/attenuated	Mitochondrial protein import and organelle homeostasis
LONP1	Lon peptidase 1, mitochondrial	Increased	Normalized	Mitochondrial protein quality control
G6PD	Glucose-6-phosphate dehydrogenase	Decreased	Restored	NADPH generation and antioxidant defense
PRDX3	Peroxiredoxin 3	Decreased	Restored	Mitochondrial peroxide detoxification
PRDX2	Peroxiredoxin 2	Increased	Normalized	Oxidative stress-responsive redox control
GSTT1A	Glutathione S-transferase theta 1a	Increased	Normalized	Detoxification and stress adaptation
FTHL27	Ferritin heavy chain-like 27	Increased	Normalized	Iron handling and oxidative stress-associated response
PARK7	Protein deglycase DJ-1	Increased	Partially restored/attenuated	Oxidative stress defense and Parkinsonism-associated neuroprotection
PSMC5	26 S proteasome regulatory subunit ATPase 5	Decreased	Restored	Proteasome assembly and protein turnover
PSMB10	Proteasome subunit beta type-10	Increased	Normalized	Proteasomal stress response
PSMC1B	26 S proteasome regulatory subunit ATPase 1b	Increased	Normalized	ATP-dependent protein degradation
PSMB3	Proteasome subunit beta type-3	Altered	Partially restored/attenuated	Core proteasome function and protein quality control
STIP1	Stress-induced phosphoprotein 1	Decreased	Restored	Co-chaperone activity and protein folding support

^a^‘Altered’ indicates that the protein showed a detectable change in MPP^+^-challenged larvae that did not consistently meet the directional threshold for unequivocal classification as increased or decreased, showing variability across biological replicates. The directional response to BjPro-7a treatment was nonetheless consistent with a rescue-associated shift and is reported accordingly

Representative proteins are organized according to their alteration in intoxicated larvae, their directional response to BjPro-7a treatment, and their main biological relevance within the rescue-associated framework

Overlap analysis among the four groups was used to generate the global proteomic overview. Functional enrichment analysis of proteins reduced by MPP^+^ and shifted upward by BjPro-7a was performed in STRING. Protein–protein interaction analysis was also conducted in STRING, and representative rescue-associated proteins were organized by Markov Cluster Algorithm clustering to identify biologically coherent modules related to mitochondrial/bioenergetic homeostasis, redox/stress adaptation, proteostasis, and synaptic/vesicular function [31]. For functional annotation and pathway interpretation, protein identities were mapped to *Homo sapiens* UniProt entries based on sequence homology, and the corresponding *Danio rerio* orthologs were considered where applicable.

### Statistical Analysis

Behavioral data are presented as boxplots. Global locomotor comparisons among the four groups and analyses of the light/dark assay were performed by analysis of variance followed by Tukey’s multiple comparisons test, according to the structure of each dataset. For light/dark cycle analyses, the previously standardized workflow employed two-way analysis of variance followed by Tukey’s multiple-comparison test [29]. Statistical significance was set at *p* < 0.05.

## Results

### BjPro-7a Reverses Basal Locomotor Deficits Induced by MPP⁺ in Zebrafish Larvae

**Fig. 1:**
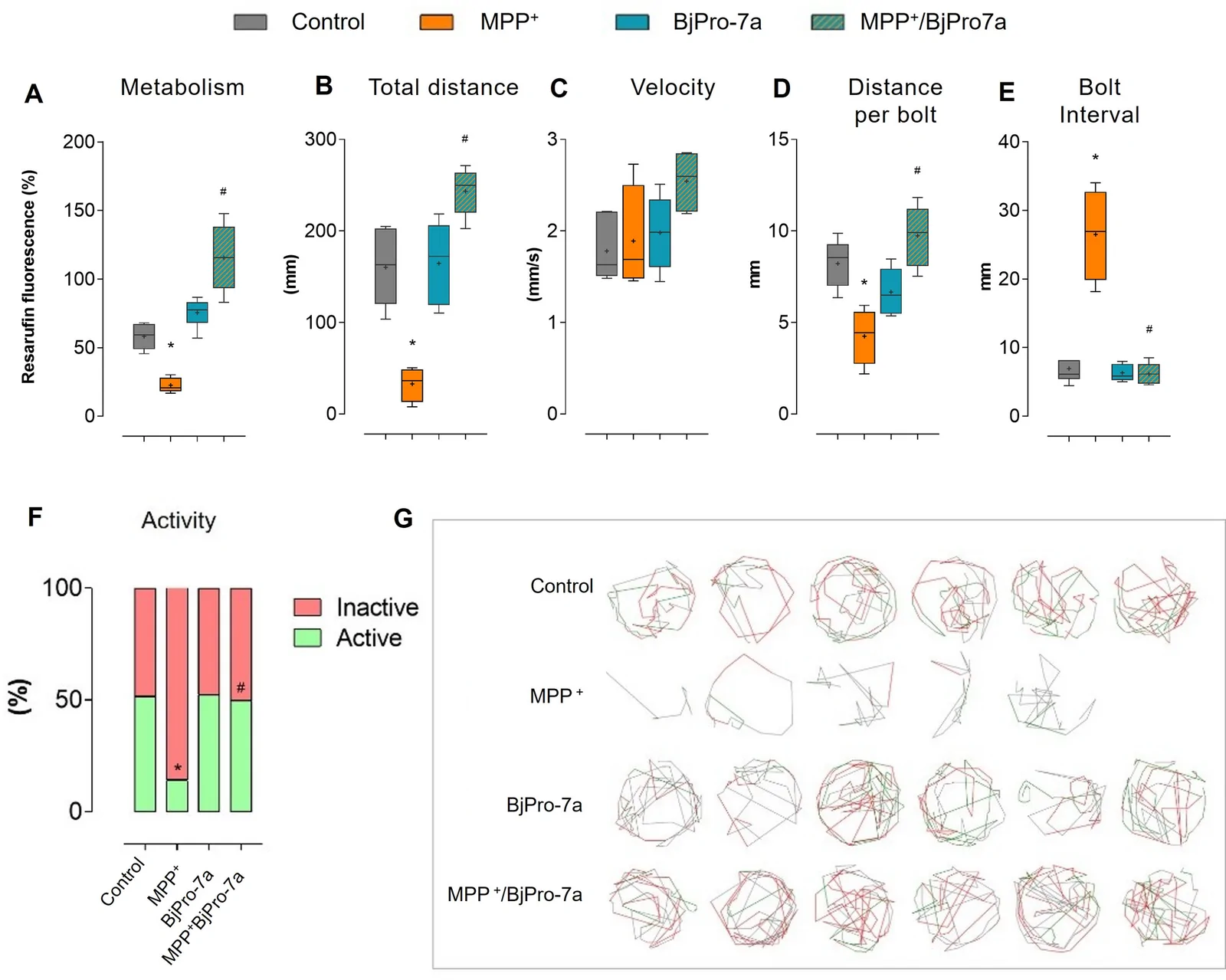
**A** Metabolic activity (resazurin reduction). **B** Total distance traveled. **C** Mean velocity. **D** Distance per bout. **E** Inter-bout interval. **F** Representative swimming trajectories. (G) Activity state. Parameters were evaluated in Control, MPP^+^, BjPro-7a, and MPP^+^/BjPro-7a groups. MPP^+^ exposure significantly reduced metabolic activity and induced a pronounced hypolocomotor phenotype. BjPro-7a alone did not alter basal metabolic or behavioral parameters. Notably, BjPro-7a treatment after MPP^+^ exposure mitigated both the metabolic deficit and locomotor impairments induced by MPP^+^. Data are presented as box plots (*n* = 12 larvae per group). Statistical comparisons were performed using one-way ANOVA followed by Tukey’s multiple comparisons test. *p < 0.05 vs. Control; #*p* < 0.05 vs. MPP^+^

Metabolic activity, assessed by resazurin reduction, was significantly impaired following MPP^+^ exposure (Fig. 1A), indicating a marked decrease in cellular metabolic function in zebrafish larvae. In contrast, larvae treated with BjPro-7a alone exhibited metabolic levels comparable to those of the Control group, suggesting that the peptide does not interfere with basal metabolic activity. Importantly, post-intoxication treatment with BjPro-7a attenuated the MPP^+^-induced metabolic deficit, restoring resazurin reduction to values approaching those observed in controls. In parallel with these metabolic alterations, exposure to MPP^+^ induced a pronounced hypolocomotor phenotype in zebrafish larvae (Fig. 1B and G). Relative to the Control group, intoxicated larvae showed a marked reduction in total distance traveled (Fig. 1B) and distance per bout (Fig. 1D), together with an increase in the interval between locomotor episodes (Fig. 1E). Mean velocity was less affected than the other endpoints (Fig. 1C), indicating that the MPP^+^-induced phenotype was more strongly associated with reduced locomotor engagement and impaired bout organization than with a complete inability to generate movement.

Larvae treated with BjPro-7a alone displayed a locomotor profile comparable to that of the Control group across all evaluated parameters. In contrast, larvae in the MPP^+^/BjPro-7a group exhibited clear attenuation of the deficits induced by intoxication. BjPro-7a treatment after MPP^+^ exposure increased total distance traveled and distance per bout and reduced the interval between locomotor episodes relative to the MPP^+^ group. The proportion of time spent in the active state was also reduced by MPP^+^ and shifted toward control values in the presence of BjPro-7a (Fig. 1G). Representative swimming trajectories were consistent with these quantitative data (Fig. 1F). Control and BjPro-7a groups showed broader and more organized exploratory paths, whereas MPP^+^-treated larvae displayed short and spatially restricted trajectories. In the MPP^+^/BjPro-7a group, this restricted pattern was substantially attenuated, with trajectories distributed more similarly to those observed in non-intoxicated larvae. Together, these findings show that BjPro-7a reverses the basal locomotor dysfunction induced by MPP^+^ in zebrafish larvae.

### BjPro-7a Restores Light/Dark-Evoked Locomotor Responses in MPP⁺-Exposed Larvae

**Fig. 2:**
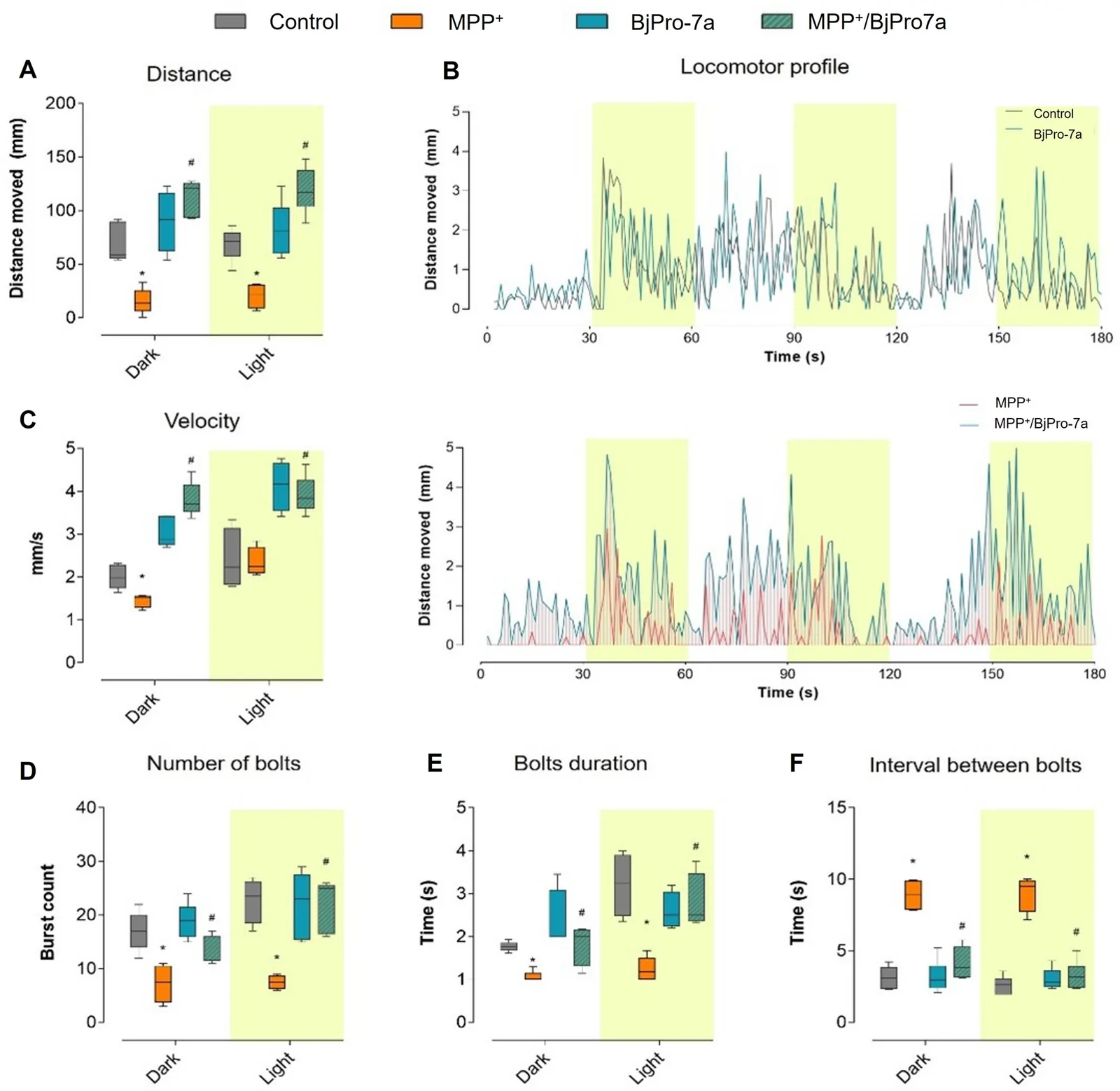
BjPro-7a restores light/dark-evoked locomotor responses in MPP^+^-exposed zebrafish larvae. Panels **A–F** show locomotor parameters recorded during alternating dark and light phases, including distance moved, velocity, bout-related measurements, and interval between movement episodes. Panel B shows temporal locomotor traces illustrating the behavioral profile throughout the assay. MPP^+^ markedly reduced locomotor responsiveness, whereas BjPro-7a preserved performance and attenuated the deficits observed in intoxicated larvae. Shaded areas indicate light phases. Data are presented as box plots (*n* = 12 larvae per group). Statistical comparisons were performed using two-way ANOVA followed by Tukey’s multiple comparisons test. *p < 0.05 vs. Control; #*p* < 0.05 vs. MPP^+^

To determine whether the therapeutic effect of BjPro-7a extended beyond spontaneous locomotion, larvae were subjected to the light/dark locomotor assay (Fig. 2A–F). Exposure to MPP^+^ reduced distance moved (Fig. 2A) and velocity (Fig. 2B) during the alternating phases of the test, in parallel with a decrease in the number (Fig. 2C) and duration (Fig. 2D) of locomotor bouts and an increase in the interval between movement episodes (Fig. 2E). Thus, MPP^+^ impaired not only overall locomotor output, but also the temporal organization of stimulus-evoked motor responses.

BjPro-7a alone preserved a robust locomotor profile throughout the assay and did not produce evident behavioral impairment. More importantly, larvae in the MPP^+^/BjPro-7a group displayed a clear improvement relative to intoxicated larvae, with higher displacement and velocity, greater number and duration of locomotor bouts, and shorter intervals between episodes. These changes indicate recovery of both movement magnitude and bout structure after treatment with BjPro-7a.

Temporal locomotor traces reinforced this pattern (Fig. 2G). Control and BjPro-7a groups exhibited preserved oscillatory activity across successive dark and light periods, whereas the MPP^+^ group showed consistently blunted responses throughout the assay. In contrast, BjPro-7a treatment after MPP^+^ exposure restored a more dynamic response profile, with activity peaks approaching those observed in non-intoxicated larvae. Taken together, these results show that BjPro-7a not only improves basal locomotion, but also restores visually evoked locomotor responsiveness in MPP^+^-challenged zebrafish larvae.

### Global Overview of Proteomic Profiling Across Experimental Groups

**Fig. 3:**
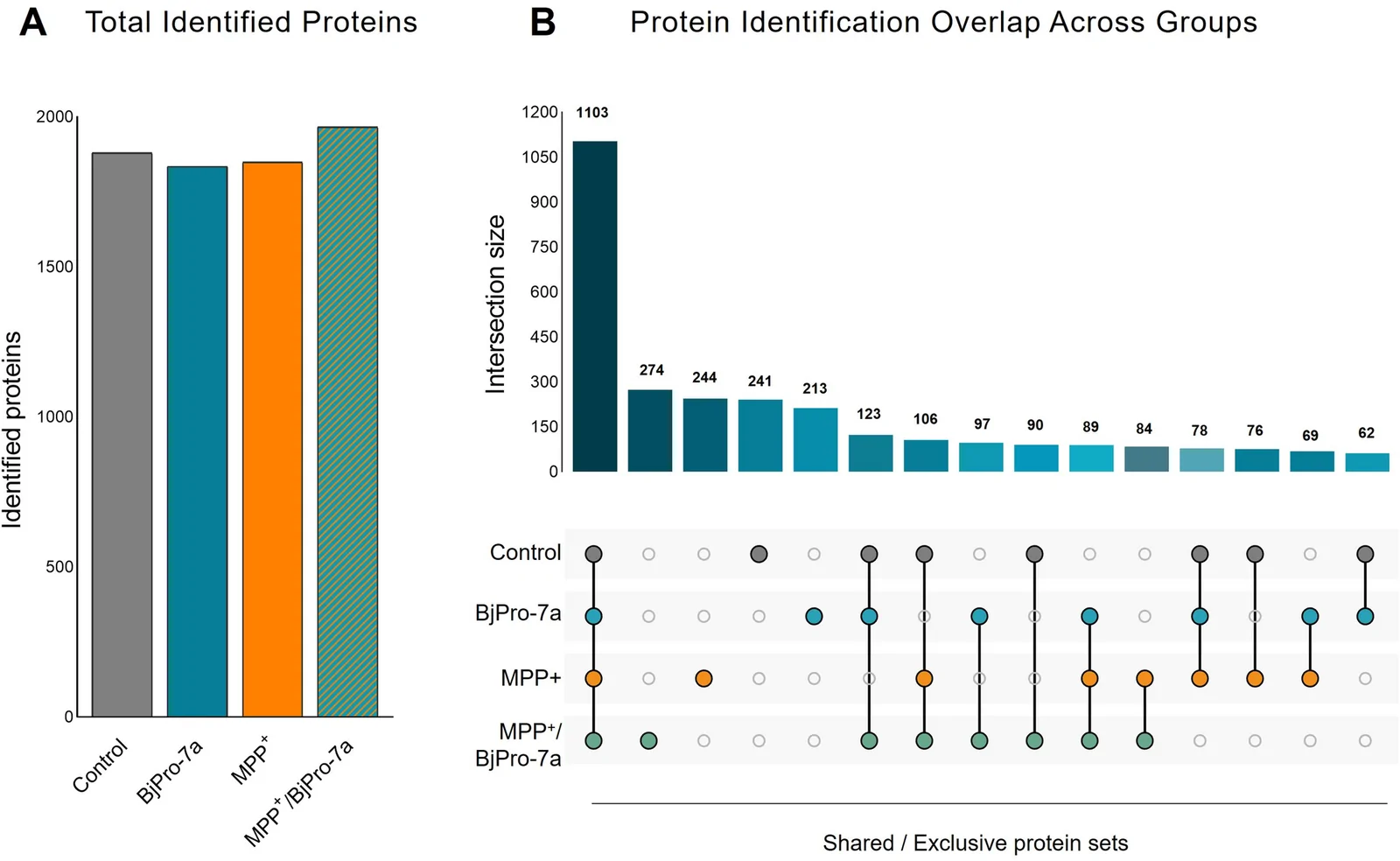
Overview of proteomic profiling across Control, BjPro-7a, MPP^+^, and MPP^+^/BjPro-7a groups. **A** Total number of proteins identified in each experimental group. Comparable proteomic coverage was observed across all conditions, with 1879 proteins detected in the Control group, 1834 in the BjPro-7a group, 1849 in the MPP^+^ group, and 1966 in the MPP^+^/BjPro-7a group. **B** UpSet plot showing the overlap of protein identifications among the four groups. A total of 1103 proteins were shared across all conditions, indicating the presence of a robust core proteome, whereas condition-specific identifications were also observed for each group

A global inspection of the proteomic dataset showed comparable protein identification coverage across all experimental groups (Fig. 3A). A total of 1879 proteins were detected in the Control group, 1834 in the BjPro-7a group, 1849 in the MPP^+^ group, and 1966 in the MPP^+^/BjPro-7a group. Thus, all conditions yielded robust proteomic depth, and peptide treatment in intoxicated larvae did not reduce dataset complexity.

Overlap analysis across the four groups revealed a substantial shared core proteome (Fig. 3B). A total of 1103 proteins were detected in all conditions, whereas each group also presented condition-specific identifications, including 241 proteins exclusive to Control, 213 exclusive to BjPro-7a, 244 exclusive to MPP^+^, and 274 exclusive to MPP^+^/BjPro-7a. These data indicate that, although the experimental conditions retained a broad common proteomic background, each treatment condition was also associated with a distinct detection profile.

The overlap pattern also showed intermediate intersections of biological interest. Some proteins were shared by Control, BjPro-7a, and MPP^+^/BjPro-7a groups but were absent from the MPP^+^ group, whereas other subsets were shared specifically between Control and MPP^+^/BjPro-7a. Conversely, additional proteins remained shared between MPP^+^ and MPP^+^/BjPro-7a. Together, these distributions support the view that BjPro-7a does not simply erase the intoxication-associated signature, but rather promotes a broader reorganization of the proteomic landscape in challenged larvae.

### Rescue-Associated Proteomic Remodeling Induced by BjPro-7a in MPP⁺-Challenged Zebrafish Larvae

**Fig. 4:**
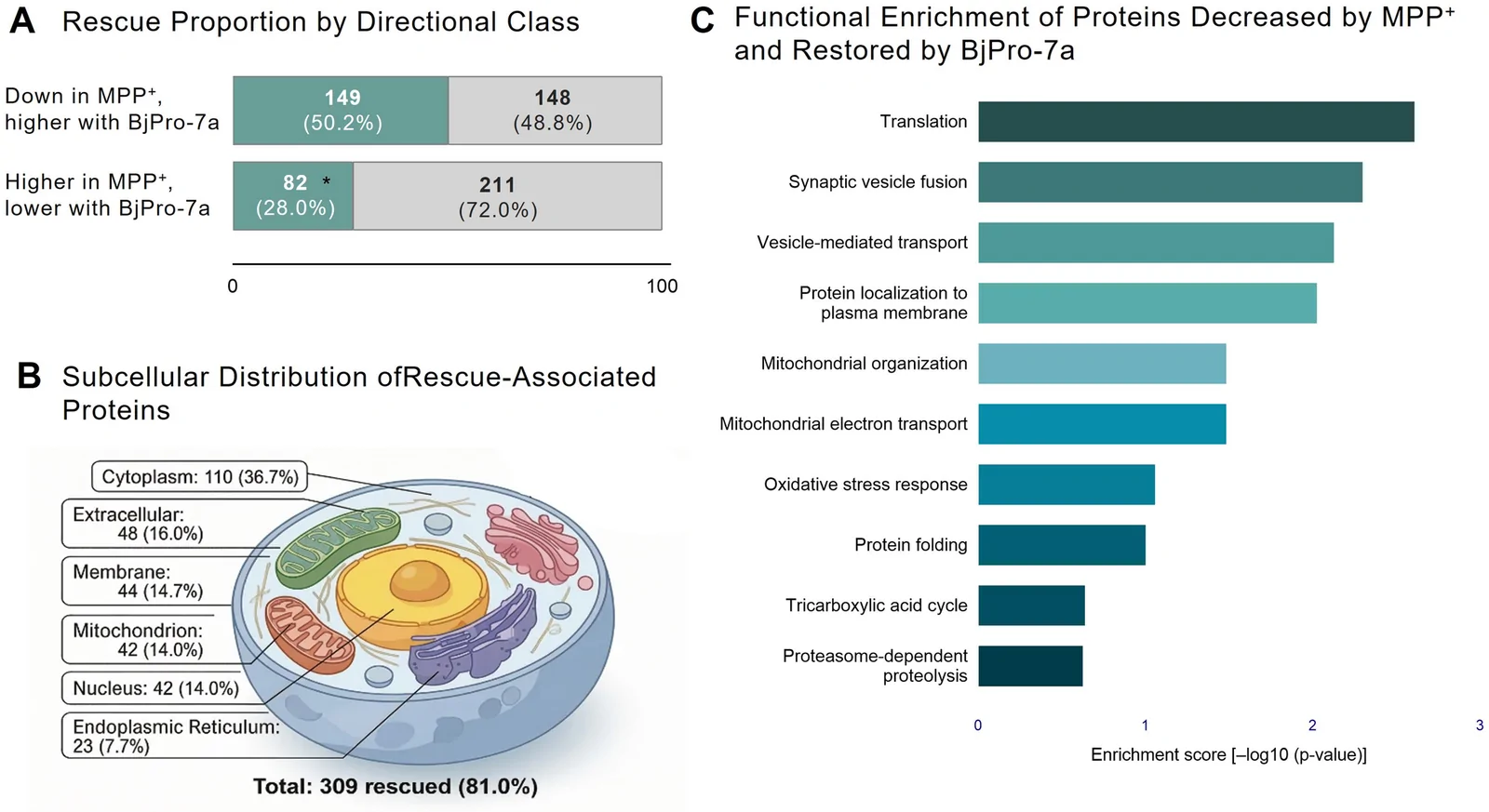
Rescue-associated proteomic remodeling induced by BjPro-7a in MPP^+^-challenged zebrafish larvae. **A** Proportion of rescue-associated proteins identified by directional comparison across experimental groups. Proteins were classified as rescue-associated when they were altered in MPP^+^ vs. Control and shifted in the opposite direction in MPP^+^ vs. MPP^+^/BjPro-7a, with the peptide-treated group moving toward the control profile. Among the proteins increased by MPP^+^, 82 of 293 (28.0%) were reduced in the presence of BjPro-7a, whereas 149 of 297 (50.2%) proteins decreased by MPP^+^ were shifted upward after peptide treatment. **B** Schematic representation of the subcellular distribution of rescue-associated proteins, showing a predominance of cytoplasmic, nuclear, membrane-associated, and mitochondrial proteins. **C** STRING-based biological process enrichment analysis of proteins decreased by MPP^+^ and shifted upward by BjPro-7a. Representative enriched terms are shown according to enrichment significance, with column height representing enrichment score and bars colored according to false discovery rate

To determine the extent to which BjPro-7a counteracted the proteomic changes induced by MPP^+^, proteins were classified according to directional reversal across the comparisons MPP^+^ vs. Control and MPP^+^ vs. MPP^+^/BjPro-7a (Fig. 4A). Rescue-associated proteins were defined as those altered in intoxicated larvae that shifted in the opposite direction after peptide treatment, with the treated group moving toward the control profile. Using this criterion, 82 of the 293 proteins increased by MPP^+^ shifted downward in the presence of BjPro-7a, whereas 149 of the 297 proteins reduced by intoxication shifted upward after peptide treatment. Thus, the rescue-associated effect was quantitatively more prominent among proteins suppressed by MPP^+^ than among those elevated by the neurotoxic challenge.

Subcellular annotation of rescue-associated proteins revealed a broad intracellular distribution, with a predominance of cytoplasmic, nuclear, membrane-associated, and mitochondrial proteins (Fig. 4B). This pattern indicates that the peptide response was not confined to a single cellular compartment, but instead involved widespread remodeling of proteins related to structural, metabolic, and regulatory functions.

To further characterize the biological processes associated with peptide-mediated restoration, the subset of proteins reduced by MPP^+^ and shifted upward by BjPro-7a was subjected to STRING-based enrichment analysis (Fig. 4C). The most significantly enriched terms were related to translational machinery, RNA-associated processes, aerobic respiration, protein folding, and metabolic homeostasis. These enriched categories point to recovery of central programs involved in biosynthetic competence, energy metabolism, and adaptive protein maintenance.

**Fig. 5:**
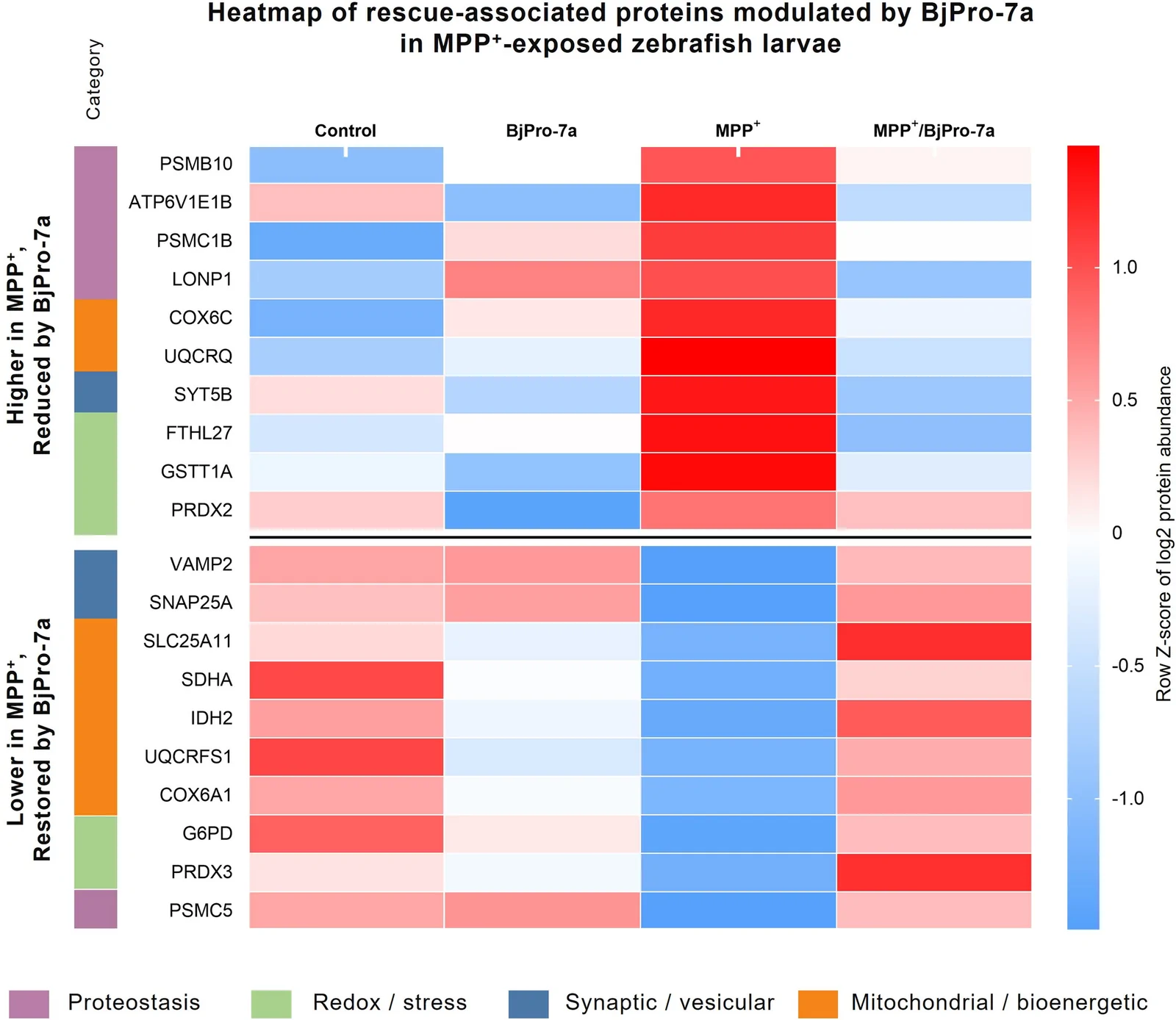
Representative rescue-associated proteins modulated by BjPro-7a in MPP^+^-challenged zebrafish larvae. Heatmap showing representative proteins displaying rescue-associated expression patterns across the Control, BjPro-7a, MPP^+^, and MPP^+^/BjPro-7a groups. Proteins were organized into two major directional response blocks: proteins increased by MPP^+^ and reduced after BjPro-7a treatment, and proteins decreased by MPP^+^ and shifted upward in the presence of the peptide. Color intensity represents row-scaled log_2_ protein abundance (Z-score). Representative proteins were grouped according to major functional categories, including synaptic/vesicular function, mitochondrial and bioenergetic pathways, redox/stress-related proteins, and proteostasis-associated factors

A heatmap of representative rescue-associated proteins further illustrated the bidirectional nature of this response (Fig. 5). One block comprised proteins elevated in intoxicated larvae and reduced in the presence of BjPro-7a, including factors associated with proteostasis, stress response, and mitochondrial regulation, such as PSMB10 and PSMC1B (proteasome function), LONP1 (mitochondrial protein quality control), ATP6V1E1B (vesicular acidification/ATPase function), COX6C and UQCRQ (mitochondrial respiratory chain), and PRDX2 and GSTT1A (redox-related defense). SYT5B, a vesicle-associated protein, also followed this pattern. The second block comprised proteins reduced by MPP^+^ and shifted upward by peptide treatment, including VAMP2 and SNAP25A (vesicle fusion and synaptic organization), SLC25A11, SDHA, IDH2, UQCRFS1, and COX6A1 (mitochondrial and bioenergetic function), G6PD and PRDX3 (redox homeostasis), and PSMC5 (proteostasis).

This integrated pattern is consistent with the functional recovery observed at the behavioral level. Rather than acting through a single isolated target, BjPro-7a appears to rebalance interconnected pathways required for cellular energetics, vesicle-associated function, oxidative adaptation, and protein quality control under neurotoxic stress. Table 1 summarizes representative proteins according to their directional response pattern and biological relevance, organizing them into restored, normalized, and partially restored/attenuated categories.

### Network Analysis Reveals Functionally Connected Modules Underlying BjPro-7a-Mediated Rescue

**Fig. 6:**
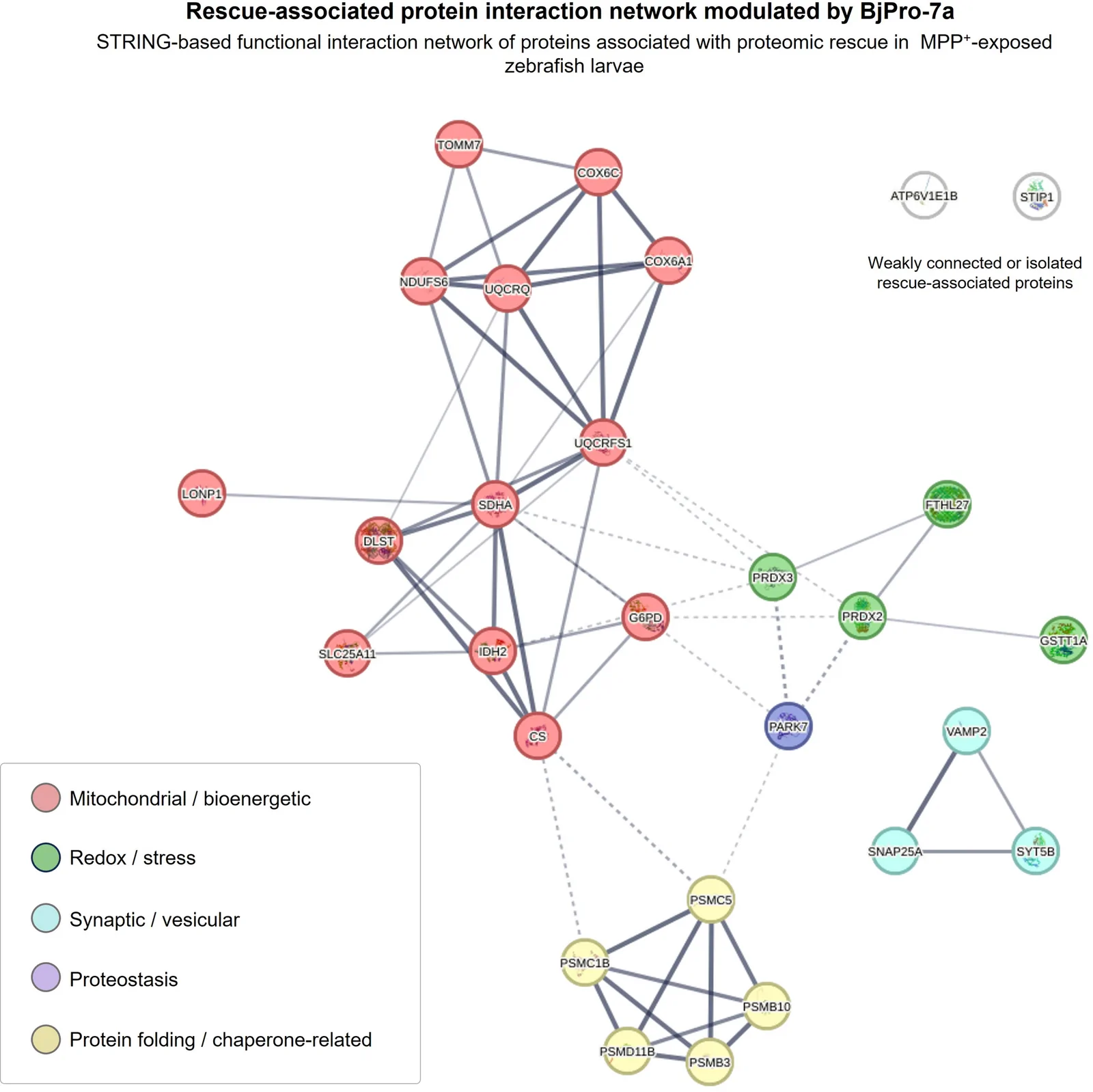
Network analysis of rescue-associated proteins modulated by BjPro-7a in MPP^+^-challenged zebrafish larvae. Protein–protein interaction network of rescue-associated proteins generated in STRING and organized by Markov Cluster Algorithm (MCL) clustering. Distinct functionally coherent modules were identified, including a central mitochondrial/bioenergetic core (SDHA, UQCRFS1, UQCRQ, COX6A1, COX6C, NDUFS6, TOMM7, CS, DLST, IDH2, SLC25A11, LONP1), a proteostasis-associated module (PSMC5, PSMC1B, PSMB10, PSMB3, PSMD11B), a redox/stress-related branch (PRDX2, PRDX3, G6PD, PARK7, FTHL27, GSTT1A), and a smaller synaptic/vesicular module (VAMP2, SNAP25A, SYT5B). Edges indicate evidence-supported interactions; node clusters reflect MCL-defined functional groups

To further investigate the relationships among representative rescue-associated proteins, a protein–protein interaction network was generated using STRING and organized by Markov Cluster Algorithm (MCL) clustering (Fig. 6). This analysis revealed a structured interaction landscape composed of biologically coherent modules, indicating that the proteins shifted by BjPro-7a were not distributed as isolated targets, but instead formed an integrated network associated with rescue.

A prominent central module was composed mainly of mitochondrial and bioenergetic proteins, including SDHA, UQCRFS1, UQCRQ, COX6A1, COX6C, NDUFS6, TOMM7, CS, DLST, IDH2, SLC25A11, and LONP1. A second functionally connected module comprised proteostasis-associated proteins such as PSMC5, PSMC1B, PSMB10, PSMB3, and PSMD11B. In parallel, a redox/stress-related branch linked PRDX2, PRDX3, G6PD, PARK7, FTHL27, and GSTT1A to the broader mitochondrial core. A smaller synaptic/vesicular module containing VAMP2, SNAP25A, and SYT5B highlighted recovery of proteins involved in vesicle dynamics and neurotransmission-related organization.

Taken together, the network architecture places mitochondrial and bioenergetic remodeling at the center of the peptide-associated response, while also showing coordinated links with proteostasis, redox balance, and synaptic/vesicular function. This systems-level organization is consistent with the behavioral recovery observed in peptide-treated larvae and provides an integrated framework connecting proteomic remodeling with functional rescue in the MPP^+^ zebrafish model.

## Discussion

The present study demonstrates that BjPro-7a markedly attenuates the behavioral and proteomic disturbances induced by MPP^+^ in zebrafish larvae, supporting a therapeutic effect of the peptide in an early Parkinsonian-like context. Our previous work established that chronic MPP^+^ exposure in zebrafish larvae produces a reproducible hypolocomotor phenotype accompanied by proteomic alterations related to mitochondrial dysfunction, oxidative imbalance, proteostasis, and synaptic biology [29]. In the current study, we extend that framework by showing that BjPro-7a not only improves locomotor performance, but also reshapes the intoxication-associated proteomic landscape in a direction consistent with rescue. This progression from injury characterization to rescue-oriented remodeling is especially relevant because zebrafish neurotoxicity models become substantially more informative when behavioral outputs and molecular endpoints converge on a biologically coherent interpretation [23–29, 32].

At the behavioral level, the pattern observed here is highly relevant when interpreted in light of Parkinsonian motor dysfunction. Larval zebrafish do not reproduce the full clinical picture of PD in a literal one-to-one manner, but toxin-induced reductions in total distance traveled, bout frequency, locomotor engagement, and visually evoked responsiveness are widely accepted as meaningful proxies of hypokinetic/bradykinetic-like impairment in this model [23–29, 32–34]. In this sense, the MPP^+^-induced phenotype observed here is not simply a reduction in spontaneous movement. It reflects a state of reduced motor drive, impaired episode initiation, and blunted responsiveness to environmental stimulation. BjPro-7a reversed this profile by increasing total displacement, improving bout organization, shortening intervals between movement events, and restoring activity levels under both basal and light/dark conditions. This pattern supports genuine functional recovery rather than a trivial nonspecific increase in movement.

The proteomic data reinforce this interpretation. One of the clearest findings of the present study is that rescue-associated remodeling was quantitatively more pronounced among proteins reduced by MPP^+^ than among those elevated by intoxication. This is biologically meaningful because neurotoxic states often involve both gain-of-stress signatures and loss of essential homeostatic functions [1–8, 35–40]. In our dataset, BjPro-7a appeared particularly effective at shifting upward proteins linked to fundamental cellular programs that had been suppressed by MPP^+^, including mitochondrial metabolism, antioxidant regulation, vesicle-associated organization, and protein quality control. Thus, the peptide response was not limited to damping stress-associated signals, but also involved restoration of functions required for a more preserved cellular state.

The mitochondrial and bioenergetic axis emerged as a central component of this response. Several representative rescue-associated proteins belonged to core mitochondrial pathways, including SDHA, UQCRFS1, UQCRQ, COX6A1, COX6C, IDH2, DLST, CS, SLC25A11, and TOMM7. These proteins collectively map onto respiratory chain activity, tricarboxylic acid cycle function, metabolite exchange, and mitochondrial organization. Given the well-established role of MPP^+^ in disrupting mitochondrial respiration and redox homeostasis, recovery of these proteins is highly consistent with reversal of a central pathogenic axis in Parkinsonian neurotoxicity [3, 4, 6, 8, 35–37]. The network analysis further supported this interpretation by placing mitochondrial and bioenergetic proteins at the core of the interaction map, suggesting that this module may represent the main molecular backbone of the rescue response.

A second major component involved redox adaptation and stress management. G6PD, PRDX3, PRDX2, GSTT1A, FTHL27, and PARK7 were among the proteins linked to this axis. These factors are functionally related to peroxide detoxification, redox buffering, glutathione-associated defense, iron handling, and broader cellular adaptation to oxidative stress. Because redox imbalance is tightly coupled to mitochondrial dysfunction in MPP^+^-based models, the fact that these proteins appeared integrated with the mitochondrial cluster is particularly relevant [3, 4, 6, 8, 35–37]. Rather than representing an independent antioxidant layer, the redox response observed here appears to be part of a broader restoration of mitochondrial–redox coupling under intoxication.

Proteostasis also emerged as an important dimension of the peptide-associated response. Proteasome-related proteins such as PSMC5, PSMC1B, PSMB10, PSMB3, and PSMD11B, together with LONP1 and STIP1-related functions, place protein quality control within the rescue-associated framework. This is important because neurodegenerative phenotypes are not sustained only by energetic collapse or oxidative stress, but also by failure to maintain proper protein turnover, folding, and surveillance [5, 38–40]. The present data suggest that BjPro-7a acts within this broader homeostatic space, shifting the proteomic profile toward a more balanced state across systems that are functionally interdependent rather than isolated.

The synaptic/vesicular dimension of the response is also noteworthy. VAMP2, SNAP25A, and SYT5B were among the representative proteins shifted by peptide treatment. These molecules are related to vesicle fusion, synaptic machinery, and vesicle-associated organization, and their modulation is particularly relevant because motor behavior ultimately depends on preserved neuronal communication and output coordination [33, 34, 41, 42]. In this context, recovery of synaptic/vesicular proteins provides a plausible molecular bridge linking proteomic remodeling with the restoration of locomotor performance observed in the behavioral assays.

An important mechanistic framework for interpreting these findings is the previously described ability of BjPro-7a to activate M1 muscarinic acetylcholine receptors [18–22]. This background is highly relevant because M1-linked signaling is associated with neuronal excitability, intracellular signaling cascades related to adaptive stress responses, and maintenance of metabolically active neuronal states [21, 22, 43–46]. In this context, the behavioral rescue and coordinated proteomic remodeling observed here are biologically consistent with a receptor-linked therapeutic effect involving restoration of synaptic, mitochondrial, redox, and proteostatic homeostasis under MPP^+^ challenge. Although receptor dependence was not directly tested in the present zebrafish model, the known pharmacological profile of BjPro-7a provides a plausible mechanistic bridge between previous in vitro and rodent observations and the systems-level rescue documented here.

Another important point is that the response to BjPro-7a did not correspond to a simple return to a naive state. The overlap analysis showed that the peptide-treated intoxicated group shared proteins both with Control and with MPP^+^ groups, while also presenting condition-specific detections. This suggests that rescue occurs through partial restoration combined with treatment-associated remodeling rather than complete erasure of the intoxication signature. Such a pattern is biologically plausible in complex in vivo systems and is fully compatible with the directional categories adopted here, namely restored, normalized, and partially restored/attenuated proteins.

These findings should be interpreted within the scope of the present experimental design. The choice of the larval zebrafish model warrants explicit consideration. Although adult organisms present a fully mature nervous system, the larval stage offers several complementary and operationally distinct advantages that justify its use at this phase of investigation. At 5 dpf, zebrafish larvae have completed the basic morphogenesis of their nervous system, including differentiation of dopaminergic populations, establishment of motor circuits, and expression of conserved components of mitochondrial and proteostatic machinery [23–26]. The larval platform further enables high-throughput experimental designs, minimizes the number of animals required per condition, allows direct pharmacological access, and yields quantifiable locomotor readouts that parallel hypokinetic features of Parkinsonian neurotoxicity. The goal of this study was to establish whether BjPro-7a produces a meaningful rescue effect and to characterize the associated molecular remodeling, objectives for which the larval model is well-suited. Evaluating the peptide in adult organisms with a more mature nervous system represents an important next step and is acknowledged as a priority for future work. Because proteomic profiling was performed using whole-larva samples, the observed changes reflect organism-level remodeling rather than cell-type-specific responses. In addition, the rescue classification adopted here identifies biologically relevant candidates based on directional consistency across group comparisons and should be further refined in future targeted validation studies. Even so, the combined behavioral and proteomic approach adopted here provides convergent evidence that BjPro-7a does not merely modify isolated readouts, but instead shifts the intoxication-associated state toward a more preserved functional and molecular profile.

Overall, the present results position BjPro-7a as a promising venom-derived peptide with multitarget therapeutic potential in early Parkinsonian-like neurotoxicity. Its effects were associated with coordinated rescue of locomotor performance and remodeling of molecular systems centered on mitochondrial function, redox adaptation, proteostasis, and vesicle-associated biology. This integrated response strengthens the view that venom-derived peptides may represent valuable scaffolds for future mechanistic and translational studies in neurodegeneration [9–13, 47, 48].

## Conclusions

In summary, the present study demonstrates that BjPro-7a attenuates both the behavioral and proteomic disturbances induced by MPP^+^ in zebrafish larvae, supporting its relevance as a multitarget therapeutic candidate in an early Parkinsonian-like context. The peptide restored basal locomotion, improved light/dark-evoked responsiveness, and promoted broad proteomic remodeling consistent with rescue of core cellular systems disrupted by intoxication. Integrative analyses indicated that this effect was associated with coordinated modulation of proteins and pathways linked to synaptic/vesicular function, mitochondrial and bioenergetic homeostasis, redox balance, and proteostasis.

Importantly, the combined use of behavioral phenotyping and proteomic profiling provided convergent evidence that BjPro-7a does not merely alter isolated readouts, but instead shifts the intoxication-associated state toward a more preserved functional and molecular profile. This systems-level rescue pattern strengthens the view that BjPro-7a is a compelling candidate for further mechanistic investigation in experimental models of Parkinsonian neurotoxicity and related neurodegenerative dysfunction.

## Data Availability

No datasets were generated or analysed during the current study. The complete proteomic table associated with this study has been deposited in Mendeley Data and is publicly available at https://doi.org/10.17632/y3nwmdfmkx.1.

## Abbreviations

BSA: Bovine serum albumin
CEUA: Ethics committee for animal use
CONCEA: Brazilian National Council for the control of animal experimentation
dpf: Days post-fertilization
E3: Embryo medium
hpf: Hours post-fertilization
LC–MS/MS: Liquid chromatography–tandem mass spectrometry
M1 mAChR: M1 muscarinic acetylcholine receptor
MCL: Markov cluster algorithm
MPP⁺: 1-methyl-4-phenylpyridinium
MS: Mass spectrometry
MS/MS: Tandem mass spectrometry
mzXML: Mass spectrometry markup language XML format
OG: 1-S-octyl-beta-D-thioglucopyranoside
PD: Parkinson’s disease
PPI: Protein–protein interaction
ROS: Reactive oxygen species
SEM: Standard error of the mean
STRING: Search tool for the retrieval of interacting genes/proteins
TU: Tübingen zebrafish strain
